# Affect regulation training reduces symptom severity in depression – A randomized controlled trial

**DOI:** 10.1371/journal.pone.0220436

**Published:** 2019-08-29

**Authors:** Matthias Berking, Eva Eichler, Maike Luhmann, Alice Diedrich, Wolfgang Hiller, Winfried Rief

**Affiliations:** 1 University of Erlangen-Nuremberg, Erlangen, Germany; 2 University of Cologne, Cologne, Germany; 3 Ludwig-Maximilian University Munich, Munich, Germany; 4 University of Mainz, Mainz, Germany; 5 University of Marburg, Marburg, Germany; University of Toronto, CANADA

## Abstract

Deficits in general emotion regulation skills have been shown to be associated with various mental disorders. Thus, general affect-regulation training has been proposed as promising transdiagnostic approach to the treatment of psychopathology. In the present study, we aimed to evaluate the efficacy of a general affect-regulation as a stand-alone, group-based treatment for depression. For this purpose, we randomly assigned 218 individuals who met criteria for major depressive disorder (MDD) to the Affect Regulation Training (ART), to a waitlist control condition (WLC), or to a condition controlling for common factors (CFC). The primary outcome was the course of depressive symptom severity as assessed with the Hamilton Rating Scale for Depression and the Beck Depression Inventory. Multi-level analyses indicated that participation in ART was associated with a greater reduction of depressive symptom severity than was participation in WLC (d = 0.56), whereas the slight superiority of ART over CFC (d = 0.25) was not statistically significant. Mediation analyses indicated that changes in emotion regulation skills mediated the differences between ART/CFC and WLC. Thus, the findings provide evidence for enhancing emotion regulation skills as a common mechanism of change in psychological treatments for depression. The study was registered with ClinicalTrials.gov (NCT01330485) and was supported by grants from the German Research Association (DFG; BE 4510/3-1; HI 456/6-2). Future research should compare the (cost-) efficacy of ART with that of disorder-specific interventions.

## Introduction

Enhancing patients’ emotion regulation skills has been proposed as a promising target in the treatment of various mental disorders [1–5]. Emotion regulation refers to the set of processes whereby people seek to monitor, evaluate, and redirect the spontaneous flow of their emotions in accordance with their needs, goals and contextual demands (e.g. [6]). Deficits in these skills can be linked to psychopathology in at least two ways. First, overly intense, long-lasting, and undesired affective states are major criteria for various mental disorders [7]. Second, many cognitive and behavioral symptoms of mental disorders can be conceptualized as dysfunctional attempts to regulate aversive feelings. For example, avoidance [8], binging [9], catastrophizing [10], (depressive) rumination [11], excessive sexual behavior [12], restricted eating [13], self-injury [14], somatization [15], substance use [16], worrying [17], and even delusions [18] have been proposed as attempts to cope with challenging feelings in the absence of adaptive strategies.

Empirical evidence for the hypothesis that deficits in ER contribute to the development and maintenance of a broad range of mental disorders comes from a large number of cross-sectional, prospective, and experimental studies [1, 5, 19, 20]. Based on these findings, it can be hypothesized that many mental disorders can be successfully treated with the help of interventions that focus exclusively upon enhancing general emotion regulation skills [21, 22]. Preliminary evidence for this assumption comes from studies indicating that Dialectical Behavior Therapy (DBT) [23], which explicitly teaches emotion regulation abilities, has been found to be effective in the treatment of several disorders, including borderline personality disorder [24, 25], (chronic) depression [26–28] substance use disorder [29], and eating disorders [30, 31]. Further treatments that focus on emotion regulation skills and have been shown to reduce symptoms of mental disorders include Cognitive Behavioral Therapy—Enhanced (CBT-E) [32], Emotion-Focused Cognitive Behavioral Therapy (ECBT) [33], Emotion Focused Therapy (EFT) [34, 35], Emotion Regulation Therapy (ERT) [36, 37], and the Unified Protocol for the Treatment of Emotional Disorders (UP) [38, 39–52].

However, for several reasons evidence from these studies for the hypothesis that symptom severity in various mental disorders can be reduced by strengthening patients’ emotion regulation skills is still preliminary. First, several of the afore-mentioned treatments include components that do not directly focus on emotion regulation skills. For example, DBT and CBT-E systematically teach interpersonal skills in addition to emotion regulation skills. Thus, it is unclear whether changes in interpersonal skills are responsible for positive outcomes (or whether emotion regulation training is only effective in combination with interpersonal skills training). Second, only some of the treatments focusing on emotion regulation skills have evaluated the efficacy of exclusively group-based versions of these treatments [26, 31, 43, 44–46, 50, 51], although the applicability of transdiagnostic treatments in cost-effective group settings is considered a particular strength of these approaches [53]. Finally, with the exception of EFT, all of these treatments were (originally) developed for the treatment of a specific mental disorder or a specific group of mental disorders (CBT-E: eating disorders; ECBT: anxiety disorders in children; ERT: generalized anxiety disorder; DBT: borderline personality disorder; UP: depressive and anxiety disorders). Thus, they can be assumed to require adaptation if they are used to treat a broader range of mental disorders or even psychopathological symptoms in general. Therefore, it is currently unclear whether mental disorders can be treated with emotion regulation trainings that are not custom tailored to the needs of specific diagnostic groups. Because major depression is one of the most common and most serious disorders [54], the present study aims to evaluate the efficacy of such a training with regard to reduce symptoms of depression in clinically depressed individuals.

The idea that a systematic training of general emotion regulation skills could be effective for depression is supported by various studies linking deficits in emotion regulation to depression (e.g., [55, 56–64]). However, many of the available studies exclusively use cross-sectional designs, suffer from the problem of uncontrolled confounding factors limiting the validity of longitudinal studies, or display low ecological validity because they exclusively investigate ultra-short-term effects in laboratory settings. Thus, there is a need for field-based experimental studies that evaluate the effects of manipulating emotion regulation skills on psychopathological symptoms with specific interventions.

Previous research on the efficacy of training of emotion regulation skills in depression includes a study in which a shortened version of the Affect Regulation Training (ART) [22, 65] was integrated into a 6-week CBT-based inpatient treatment of a heterogeneous clinical sample (N = 289; half of which met criteria for major depressive disorder, MDD). The results from this study provided preliminary evidence that enriching traditional CBT with interventions such as ART may further improve the efficacy of CBT for the treatment of depression (see 2 for details). Subsequently, these findings were replicated in a randomized controlled trial with 431 participants who all met the criteria for MDD [66]. However, none of these studies evaluated ART as a stand-alone intervention. Thus, it can be argued that ART only works in interaction with CBT. Moreover, because CBT also targets emotion regulation skills in a direct way (e.g., by utilizing reappraisal or behavioral activation to improve one’s mood), there is a need for studies using conditions that control for unspecific treatment effects while minimizing the use of interventions that directly target emotion regulation skills.

Thus, the primary aim of the present study was to evaluate the efficacy of an emotion regulation training that does not provide participants with disorder-specific information or training but focuses exclusively on enhancing adaptive responses to undesired affective states (such as stress/tension, anger, anxiety, sadness, dysphoric/depressed mood, shame, and guilt) with regard to the reduction of depressive symptom severity (DSS) in patients meeting criteria for MDD. To attain this goal, we conducted a randomized controlled trial in which individuals meeting criteria for MDD and seeking outpatient treatment were randomly allocated to ART, a waitlist control condition (WLC), or a condition that was developed to control for common psychotherapeutic factors (CFC). More specifically, we tested the hypothesis that ART would be more effective than WLC and CFC with regard to reducing DSS. Additionally, we tested whether ART would lead to a greater increase of emotion regulation skills and a greater reduction of general psychopathology than WLC or CFC. Finally, we tested whether a greater increase of emotion regulation skills would mediate the hypothesized superiority of ART over WLC and CFC with regard to reducing DSS.

## Materials and methods

### Design and procedure

**Fig 1:**
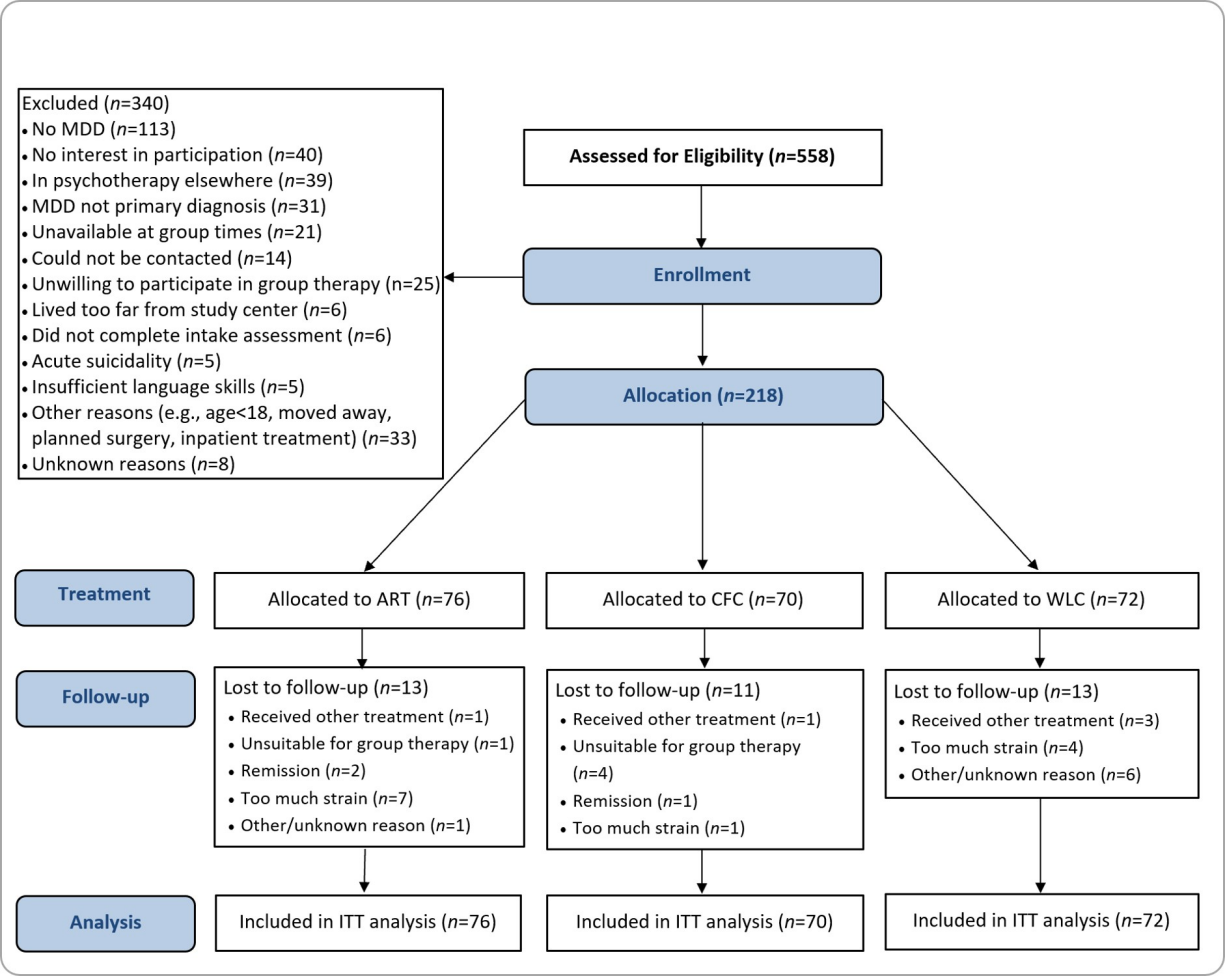
Patient flow. ART = Affect Regulation Training; CFC = common factor control condition; WLC = waitlist control condition; ITT = intention to treat.

We evaluated the efficacy of ART in a multicenter, prospective, 3-armed, randomized controlled trial (see Fig 1 for illustration). The study was conducted between November 2010 and April 2014. In addition to clarifying the stand-alone effects of a transdiagnostic training of emotion regulation skills on symptom severity in depressed patients, the trial aimed to evaluate potential augmentation effects on subsequent individual CBT for depression (findings will be reported elsewhere [67]). Participants were recruited from individuals applying for psychotherapeutic treatment at one of the three outpatient treatment centers where the study was conducted. During the intake telephone call, patients were pre-screened with a short list of questions addressing inclusion and exclusion criteria. Eligible and consenting individuals received further information about the study and were invited to an in-depth diagnostic interview. Diagnostic status was assessed through the Structured Clinical Interview for DSM-IV Axis I- and II Disorders (SCID; [68]). SCID and Hamilton Rating Scale for Depression (HRSD; [69]) interviews were conducted face-to-face by psychotherapists in advanced clinical training who had been intensively trained in both instruments (SCID: at least two days of training; HRSD: at least one day of training) and who were supervised by experienced assessors (who had been responsible for patient intake and diagnostics in the study centers for several years). Video recordings of 5% of all interviews were rated by a second assessor (intensely trained research staff responsible for conducting all clinical interviews at the outpatient treatment center of the University of Erlangen-Nuremberg, supervised by a senior psychotherapist) to evaluate inter-rater reliability (see Measures). Patients who met all of the inclusion criteria and none of the exclusion criteria were randomly assigned to ART, CFC or WLC. Block randomization was conducted by research assistants who were located at two of the three study centers and were not otherwise involved in the study. Randomization was conducted with the help of a computerized randomization tool (randomisation.net, P = 1/3 for each condition; bloc sizes ranging between 12 and 24) stratifying by gender, the presence of comorbid diagnoses (present vs. not present), the use of antidepressant medication (use vs. no use), and the number of previous depressive episodes (0 vs. 1 or above). All participants using antidepressant medication were asked to refrain from changing their medication during the study period, if possible.

During the course of the study, all participants were invited to complete self-report assessments of DSS, adaptive responses to undesired feelings, and general psychopathological symptom load at six assessment points (with 2-week intervals between assessment points T1 to T5 and a 4-week interval between T5 and T6). Additionally, they participated in observer-based assessments of DSS and adaptive responses to undesired feelings at T1 and T6. These measures were assessed on a regular bi-weekly schedule with the help of paper-and-pencil questionnaires that were sent to participants and returned to the treatment center by mail. Data was stored in a de-identified format on highly protected servers of the collaborating universities with only specifically trained study staff having access of the identifiers of each center. All study procedures were approved by the ethical committee of the German Psychological Society (July 31, 2010). The trial was registered with ClinicalTrials.gov, number NCT01330485. Because of administrative problems, release of registration occurred about six months after study start. The authors confirm that all ongoing and related trials for this intervention are registered. Relevant de-identified data can be downloaded from https://osf.io/426pd.

### Participants

To be included in the study, participants had to meet the following criteria: informed consent, current primary diagnosis of MDD, age 18 or above, and sufficient (German) language skills. Capacity to consent was determined by experienced psychotherapists conducting intake interviews with individuals applying for treatment at one of the study centers. Exclusion criteria included high risk of suicide, evidence of significant secondary gain (e.g., early retirement application based on mental health problems), and acute comorbid psychotic disorder, substance use disorder, bipolar disorders, organic brain disorders, or other severe medical disorders that would interfere with study procedures. To maximize the external validity of the study, there were no other exclusion criteria (e.g., other comorbid disorders, use of antidepressant medication).

### Measures

#### Diagnostics

The SCID [68] was used to assess diagnostic status at both the intake interview (T1) and the last assessment point (T6). The analyses of inter-rater reliabilities of a random sample of 5% of the SCID interviews yielded a Cohen’s kappa [70] of κ = .85, indicating very good inter-rater reliability regarding the presence of a diagnosis of MDD.

#### Primary outcome measures

The primary outcome was the rate of change of depressive symptoms as assessed by the 24-item version of the Hamilton Rating Scale for Depression (HRSD; [69]) and the revised Beck Depression Inventory (BDI-II; [71, 72]). The HRSD is a clinician-administered semi-structured interview that assesses symptoms of depression. Based on patients’ responses, clinicians rate the degree of 24 symptoms such as depressed mood, feelings of guilt, sleep disturbances, and anxiety on 3-point or 5-point Likert scales. Higher sum scores indicate greater symptom severity. The cut-off points of 10, 19, 27, and 35 represent the thresholds for mild, moderate, severe, and very severe depression, respectively. The HRSD has been shown to have good validity and reliability [73], to be sensitive to change, and to be highly correlated with overall clinical ratings of symptom severity [74, 75]. The analysis of inter-rater reliabilities in a random sample of 5% of the HRSD interviews yielded a mean intraclass correlation (ICC; [76]) of.94, indicating excellent inter-rater reliability. The HRSD also showed good internal consistencies at T1 and T6 (α_T1/T6_ = .79/.81).

The BDI-II is a commonly used self-report measure of depression with evidence-based reliability and validity [77–79]. The self-report instrument contains 21-items assessing somatic, behavioral, emotional, and cognitive symptoms of depression on a 4-point Likert-type scale. High sum scores (range: 0–63) indicate severe depression. In the present study, the BDI-II showed good internal consistencies at all assessment points (α_T1/2/3/4/5/6_ = .89/.90/.92/.92/.92/.91).

#### Secondary outcome measures

To assess self-reports of adaptive responses to challenging feelings, we used the Emotion Regulation Skills Questionnaire (ERSQ; [80]). The ERSQ is a 27-item self-report instrument that utilizes a five-point Likert-type scale (0 = *not at all* to 4 = *almost always*) to assess respondents’ adaptive emotion regulation skills in the previous week. Items are based on the adaptive coping with emotions (ACE) model [65, 81] and are grouped into the following nine subscales (each consisting of three items): *Awareness* (e.g., “I paid attention to my feelings”), *Sensations* (e.g., “My physical sensations were a good indication of how I was feeling”), *Clarity* (e.g., “I was clear about what emotions I was experiencing”). *Understanding* (e.g., “I was aware of why I felt the way I felt”), *Acceptance* (e.g., “I accepted my emotions”), *Tolerance* (e.g., “I could endure my negative feelings”), *Readiness to confront distressing situations if needed to attain personally relevant goals* (e.g., “I pursued goals that were important to me, even if I thought that doing so would trigger or intensify negative feelings”), *Self-Support* (e.g., “I supported myself in emotionally distressing situations”), and *Modification* (e.g., “I was able to influence my negative feelings”). In addition to the subscales, the ERSQ consists of a total score computed as the average of all 27 items. Good reliability and validity of the ERSQ have been demonstrated in previous studies (e.g. [2, 16, 21, 55, 57, 64, 66, 80, 82, 83]). At all assessment points of the present study, the ERSQ showed at least acceptable internal consistencies for the subscales (α = .70 - .92) and excellent internal consistency for the total score (α = .94 - .97). In an attempt to explore the validity of self-report measures for emotion regulation, we complemented the use of the ERSQ with an observer-based version (ERSQ-OB, unpublished; a copy can be obtained from the first author) at T1 and T6. In the ERSQ-OB, spouses, friends, or close relatives rate the same adaptive responses (of the study participant) to challenging feelings that are addressed in the ERSQ. However, because many depressed individuals lack close relationships, the use of this measure can be anticipated to result in a significant and non-random proportion of missing data. Thus, we only used the ERSQ-OB as a complementary measure on an exploratory basis. In the present study, the ERSQ-OB showed acceptable-to-good internal consistencies for the nine subscales (α = .70 - .89) and excellent internal consistency for the total score (α _T1/6_ = .95/.97).

To assess psychopathological symptoms other than depression, we used a modified version of the Brief Symptom Inventory (BSI) [84, 85]. The BSI contains nine subscales (*Somatization*, *Obsessive-compulsive*, *Interpersonal Sensitivity*, *Depression*, *Anxiety*, *Hostility*, *Phobia*, *Paranoia*, and *Psychoticism*), which can be summarized by several indices. To assess psychopathology other than depression, we used the average score of all items except for those that assess depression (BSI*). The BSI has been shown to have good psychometric properties [85, 86]. In the present study, the BSI* showed good internal consistencies at both assessment points (BSI*: α_T1/6_ = .95/.96).

### Treatment

#### Affect regulation training (ART)

ART is a transdiagnostic, group-based intervention that aims to enhance general emotion regulation skills in at-risk and clinical populations. The training works to strengthen adaptive responses toward a broad range of affective states (as some of these states, e.g. stress/tension, dysphoric/depressed mood, guilt, loneliness, would not be included in a narrower definition of the term “emotion” as used by some authors (e.g., [87]; pp. 3–20) we choose the term affect regulation training; however, following a broader definition of the term (e.g., [88]; pp. 117–123), we use the terms “affect regulation” and “emotion regulation” interchangeably in the present manuscript). ART synthesizes elements from a number of psychotherapeutic approaches, such as CBT [89], Dialectical Behavior Therapy [23], Emotion Focused Therapy [35], mindfulness-based interventions [90], neuro-psychotherapeutic translational approaches [91], Compassion Focused Therapy [92], Problem Solving Therapy (PST) [93], strength-focused interventions [91, 94], and Vipassana-based approaches to mental health [95].

ART is a genuinely transdiagnostic intervention in the sense that it was developed for the prevention and treatment of any form of psychopathology. In the present study, no modifications were undertaken to adapt the training to the specific topics and needs of depressed patients. ART starts with a psycho-educational module educating participants about affective states and adaptive responses toward these states. This module includes a thorough outline of the biological and psychological origins, functions, mechanisms, and possible risks and benefits of stress responses, emotions, and mood states. Integrating findings from the affective neurosciences, seven neural “vicious circles” are presented that are deemed important for long-term maintenance of undesired affective states. Building on these models, the following emotion regulation skills are introduced: (1) muscle relaxation, (2) breathing relaxation, (3) non-judgmental perception and description of one’s feelings, (4) acceptance and tolerance of undesired feelings, (5) compassionate self-support when working to cope with such feelings, (6) constructive analysis of the antecedents and consequences of one’s feelings, and (7) active modification of one’s feelings toward a desired direction.

ART strongly emphasizes the need for systematic and continuous skills practice. Patients are taught a specific set of skill-building exercises; they develop their own daily training regimen to practice the skills; they are provided with audio CDs [96] that guide them through the skill-building exercises; and they can choose to receive text messages or e-mails that suggest 140 short exercises throughout the training period. Following the guidelines for the intensive ART format [22, 65], we scheduled one 180-minute session per week for a period of six consecutive weeks followed by another four weeks of independent skills practice (with one 90-minute booster session in the 8th week). Training groups included four to eight participants. For further details on ART, see [22, 67, 81, 97].

#### Waitlist control condition (WLC)

To control for spontaneous recovery and other time-related factors, we compared changes during ART with changes during a waiting period of the same length as the ART training. Participants in the waitlist control (WLC) condition had access to standard primary care but were asked to refrain from utilizing (secondary) psychotherapeutic care. After completing all assessments, WLC participants were offered participation in the ART training or enrollment in individual psychotherapeutic treatment at one of the study centers.

#### Common factor control condition (CFC)

To help clarify whether the potential effects of ART result from the training’s specific focus on enhancing emotion regulation skill, we compared changes during ART with changes during a condition developed to control for common factors likely to foster change in various treatments [98]. Following the work of Grawe [91, 99, 100], we sought to implement the following common factors: (1) a strong working alliance between therapists and patients (and among patients in group therapy); (2) activation of patients’ strengths, capacities, and resources; (3) fostering patients’ understanding of factors maintaining their problems; (4) enhancing patients’ skills to actively solve their problems; and (5) facing problematic situations and working to apply coping skills while the neural representation of the problem is strongly activated. Again, following the work of Grawe [91, 99], we focused upon fostering patients’ insight into which (subjective) misfits of personal goals and their perception of reality contributed to their affective or behavioral problems [22]. Based on these insights, patients were taught ways of reducing this misfit, either by letting go of unattainable goals or by working to achieve attainable goals. Evidence for the validity (and the heuristic value) of this approach to psychotherapeutic treatments comes from research focusing on the origin of affective states (e.g., [101]), psychopathology in general (e.g., [102]), and applying similar frameworks to better understand and treat depression (e.g., [93, 103]).

More specifically, the group-based treatment in the common factor control condition (CFC) started with an exercise developed by Willutzki and Koban [104] in which participants imagine a “perfect day” five years in the future under the assumption that until that date, everything went perfectly according to their wishes. After a brief relaxation exercise, the patients are invited to visualize this perfect day from morning to evening. After this exercise is completed, participants analyze their experiences with the help of a schema that guides them to infer salient wishes from their imagination (e.g., visualizing oneself lounging on a pleasant beach may lead to the deduction that the patient currently suffers from too much stress and that there is a wish for more pleasant experiences and less taxing demands from the self or others). Starting with one to five identified salient wishes, the schema then guides patients to clarify to what extent these wishes are attainable and whether the patients actively work toward the wish coming true. With the help of the therapist and the other group members, patients are invited to generate goals they want to work on or identify the wish as an unrealistic dream that causes negative affect but cannot be attained (or that can only be attained at a price the patient is unwilling to pay). Patients are then invited to divide goals they want to address into more specific sub-goals, or to consider disengaging from (likely) unattainable wishes. Subsequently, patients are taught problem-solving skills to foster the attainment of realistic goals and acceptance-focused skills to foster disengagement from unattainable wishes. The latter includes information on the importance of allowing oneself to grieve over a significant loss and instructions on how to engage in effective grief work. All members of the groups are invited to work on at least one unattainable wish to practice their ability to accept and let go of unrealistic aspirations. During this process, patients are invited to write the unattainable wishes on an index card and to explain to the group why it is so difficult to let go of these wishes and why they have decided to do so nevertheless. They then fix the index card to a helium balloon and say good-bye to the wish-bearing card, which is then carried into the sky together with the cards of the other group members. Based on these skill-building exercises, patients are invited to systematically monitor goal attainment in their daily lives and respond with acceptance or problem solving to significant misfits when a significant lack of goal attainment was perceived. Given the bona fide use of evidence-based interventions based on a consistent theoretical framework [91], CFC should be considered a very strong control condition (that may be conceptualized as an alternative transdiagnostic treatment; i.e., a standardized version of Grawes “Allgemeine Psychotherapie” [91]). Another reason for using CFC as a control condition was that therapists may tend to display a preference for newer “waves” of treatments over more established ones, and that–with the ongoing discussion of common factors—a new treatment developed to systematically apply these factors (i.e., CFC) might be perceived as equally innovative as a transdiagnostic training of emotion regulation skills by study therapists. Moreover, because the primary investigator continues to be very enthusiastic about the work of Klaus Grawe, the use of a standardized version of Klaus Grawe’s approach can be assumed to reduce the risk of allegiance effects that compromise the validity of the present study. On a more critical note, it should be acknowledged that there is a certain overlap between ART and CFC (e.g., both interventions use the general problem-solving model; GPM). However, the treatments differ significantly with regard to how directly they focus on emotion regulation skills (e.g., ART uses the GPM as the basis for a step-by-step approach of replacing an undesired feeling with a less painful one, whereas CFC uses the GPM to help attain unattained goals).

#### Treatment integrity

Treatments in ART and CFC followed standardized treatment protocols (ART: [22]; CFC: an unpublished copy can be obtained from the first author). The therapists were master’s-level psychologists who had completed or were in advanced stages of their clinical training. All therapists were intensively trained in both ART (at least 3 days of training) and CFC (at least one day of training) and provided both approaches (with each group consistently being serviced by the same therapist). When delivering the training, all therapists received regular (partially video-based) supervision (20–50 minutes of supervision after every second treatment session) from experienced ART trainers or CFC therapists. In these sessions, videos from treatment sessions were used to assess adherence to the ART/CFC protocol. For this purpose, the therapist’s performance regarding the implementation of ART/CFC-specific strategies was rated on 10-point Likert scales (copies of the adherence scales can be obtained from the first author). Scores below eight were discussed with regard to how adherence could be improved. In a subsample of approximately 20% of all treatment sessions, adherence to treatment protocols was rated by an ART/CFC expert. In all of these ratings, the adherence total per session was eight or higher. ART and CFC were identical with regard to the number, length, and scheduling of group sessions.

#### Statistical analyses

To test our primary hypotheses, we used multilevel growth models with measurement occasions (Level 1) nested within individuals (Level 2). Time was coded in terms of weeks since the first measurement occasion and was included as a Level-1 variable. The intervention group was included as a Level-2 variable. In each analysis, we included both measures of depression (HRSD and BDI-II) and tested whether these measures differed with regard to changes across treatment groups. To test the hypotheses that ART would be superior to WLC/CFC with regard to the reduction of depressive symptoms, we tested the effects of treatment condition (ART vs. WLC/CFC) on symptom reduction over time as a Level-2 moderator. Main effects and interaction effects were tested with χ^2^ difference tests. These tests reflect the difference in model fit between a model that does not include a particular effect and a model that includes a particular effect. Significant cross-level interactions between time and group were followed up by computing simple slopes for each group, which reflect the average rate of change within each group. Parameters were estimated using the Restricted Maximum Likelihood (RML) method, for the comparisons of the model fit we used Full Maximum Likelihood (FML). The same method was used when testing differences between conditions with regard to emotion regulation and psychopathological symptoms other than depression. In these analyses, all variables were standardized on the pre-treatment means and standard deviations. Hence, for all outcomes, rates of change can be interpreted in standard deviation units (Cohen’s d). Additionally, we report remission rates for each group. Following suggestions provided by Rush and colleagues (2003) and the German national guideline council on the treatment of depression [105], we defined the absence of clinically relevant symptoms as a HRSQ sum score of 9 or below and as a BDI-II sum score of 12 or below.

To clarify whether enhancing adaptive responses to undesired feelings is responsible for improvements in symptoms of depression in ART, we tested whether changes in ERSQ mediated potential differences in the rate of change of DSS between ART and WLC. For this purpose, we used a path model in which the intervention group affects post-treatment emotion regulation skills (controlling for pre-treatment emotion regulation skills), which then affect post-treatment primary outcomes (controlling for pre-treatment primary outcomes). The indirect effects were tested using bias-corrected bootstrapped 95% confidence intervals (number of bootstrap draws = 1,000 [106]). Because being aware of one’s wishes and goals, letting go of unattainable goals, and actively working to pursue realistic goals can be considered an adaptive way of responding to undesired affective states, we used the same procedures to test the hypothesis that the potential superiority of CFC over WLC would also be mediated by an increase in ER skills.

We initially powered the study on the basis of the medium-to-large effect (d = 0.65) that we expected for the comparison of ART and WLC on the basis of previous research ([1, 66]; computed with G*Power). Subsequently, we were able to raise additional funding for an experimental study nested within the RCT comparing ART, CFC and WLC (DFG; BE BE4510/3-2). This funding allowed us to extent the sample size to about n = 70 per group and, hence, detect an effect size of d = 0.42 in comparisons between two groups with β = .80 and α = 0.05. All computations were performed in R 3.1.3 [107]. Multilevel models were estimated using the R package lme4 [108]. Simple slopes, model comparisons using χ^2^ difference tests, confidence intervals, and p values were computed with the packages lsmeans [109], car [110], and lmerTest [111], which use Satterthwaite-adjusted degrees of freedom. The mediation model was estimated in the R package lavaan [112].

## Results

### Sample characteristics

Out of 558 patients screened for eligibility, 218 met the study criteria and were allocated to the study conditions (see Fig 1 for more information on reasons for exclusion). Participants in the final sample were primarily female (64.2%) and had an average age of 38.9 years (SD = 12.7). More than half of the sample (55.6%) met criteria for at least one mental disorder other than depression (mostly social phobia, 12.9%, and panic disorder, 12.5%). There were no significant differences between treatment conditions with regard to sociodemographic characteristics and diagnostics. More information on the participants is presented in Table 1.

**Table 1:** Sociodemographic characteristics and comorbidity at baseline.

Baseline characteristic	indicator	Total (N = 218)	ART (n = 76)	CFC (n = 70)	WLC (n = 72)	Test-statistic	p
Gender						0.43^b^	.81
	female, n	140	49	43	48		
	female, %	64.2	64.5	61.4	66.7		
Age						2.28^a^	.11
	range	18–69	19–65	18–64	19–69		
	M	38.9	38.8	36.6	41.1		
	SD	12.7	12.7	12.5	12.8		
Marital status						12.93^b^	.23
	Single, n	47	15	17	15		
	Single, %	21.6	19.7	24.3	20.8		
	Married/partner, n	149	54	43	52		
	Married/partner, %	68.3	71	61.5	72.2		
	Divorced, n	20	7	8	5		
	Divorced, %	9.2	9.2	11.4	6.9		
	Other, n	1	0	1	0		
	Other, %	0.5	0	1.4	0		
Highest educational level						3.22^b^	.98
	No graduation, n	4	1	1	2		
	No graduation, %	1.8	1.3	1.4	2.8		
	Middle school, n	38	13	13	12		
	Middle school, %	17.4	17.1	18.6	16.7		
	Secondary school, n	68	24	19	25		
	Secondary school, %	31.2	31.6	27.1	34.7		
	Upper second. school, n	69	26	22	21		
	Upper second. school, %	31.6	34.2	31.4	29.1		
	University, n	38	11	15	12		
	University, %	17.4	14.5	21.4	16.7		
Comorbid diagnoses						3.67^b^	.72
	1, n	85	28	29	28		
	1, %	39	36.8	41.4	38.9		
	2, n	26	10	8	8		
	2, %	11.9	13.2	11.4	11.1		
	≥ 3, n	10	1	4	5		
	≥ 3, %	4.6	1.3	5.7	6.9		
Frequent comorbid diagnoses						43.15^b^	.51
	Social phobia, n	28	11 (	10	7		
	Social phobia, %	12.9	14.4)	14.3)	9.7		
	Panic disorder, n	27	10	7	10		
	Panic disorder, %	12.5	13.2	10.0	13.9		
	Dysthymia, n	21	7	5	9		
	Dysthymia, %	9.6	9.2	7.1	12.5		
	Eating disorders, n	18	3	7	7		
	Eating disorders, %	8.4	3.9	10.0	11.1		
	Specific phobia, n	16	6	5	5		
	Specific phobia, %	7.3	7.9	7.1	7.0		
Anti-depressant medication						0.78^b^	.68
	Yes, n	61	20	23	18		
	Yes, %	28.0	26.3	32.9	25.0		

ART = Affect Regulation Training; CFC = Common Factor Control Dondition; WLC = Waitlist Control Condition

^a^ = ANOVA

^b^ = χ^2-^Test.

### Preliminary analyses

As shown in Fig 1, of 558 individuals applying for treatment at one of the study centers, 218 met the study criteria and were randomly allocated to one of the three treatment conditions. There were no significant differences across groups regarding sociodemographic data, number of comorbid diagnoses, or use of antidepressant medication, or with regard to any of the outcome measures at baseline (see Tables 1 and 2). Only a small number (n = 14) of patients reported changes in medication (ART: n = 5; CFC: n = 6; WLC: n = 3). Residual plots for homoscedasticity and QQ-Plots for normal distribution of the residuals indicated that assumptions associated with our statistical approach were met. As random slopes were significant for the time variables, indicating significant interindividual differences in the trajectories, we retained random slopes in all multilevel growth models. In all groups, DSS decreased significantly during the assessment period (pre-post effect sizes for ART/CFC/WLC: d = -0.78/-0.52/-0.22; see Table 3 for details). A non-significant three-way interaction between time, group, and outcome measure, χ^2^(2) = 0.71, p = .70, indicates that the rate of change in the three intervention groups did not differ significantly between the two measures for the primary outcome (i.e., HRSD and BDI-II). In subsequent analyses of DSS, we included the type of outcome measure (HRSD vs. BDI-II) as a Level 1 covariate. Because this covariate did not interact with any other variables, the other effects in the model can be interpreted as average effects across both measures. We included both measures in all subsequent analyses to enhance the statistical reliability. A significant two-way interaction between time and group in an omnibus test simultaneously comparing all three conditions showed that rates of change of DSS, χ^2^(2) = 14.90, p < .001, and change of emotion regulation skills, ERSQ: χ^2^(2) = 14.27, p < .01; ERSQ-OB: χ^2^(2) = 10.83, p < 0.01, differed significantly across all conditions. With regard to psychopathological symptoms other than depression, this omnibus test failed to reach statistical significance by a narrow margin, BSI*: χ^2^(2) = 5.38, p = .07.

**Table 2:** Descriptive statistics for all measures by group at all assessment points.

Outcome	Group	T1			T2		T3		T4		T5		T6	
		M (SD)	n	F (p)	M (SD)	n	M (SD)	n	M (SD)	n	M (SD)	n	M (SD)	n
HRSD				0.49 (.62)	-		-		-		-			
	ART	21.77 (8.04)	66										14.87 (8.23)	62
	CFC	20.59 (8.05)	61										15.56 (9.21)	55
	WLC	20.44 (9.00)	61										17.68 (8.90)	53
BDI-II		-		1.79 (.17)	-		-		-		-		-	
	ART	28.85 (10.32)	68		24.82 (11.17)	66	21.84 (11.43)	61	21.14 (10.94)	58	19.71 (9.73)	52	19.76 (11.03)	54
	CFC	26.61 (10.01)	53		23.61 (9.78)	57	21.11 (10.84)	55	20.00 (12.19)	46	22.48 (12.12)	42	20.58 (10.73)	50
	WLC	25.27 (11.25)	52		22.09 (10.24)	57	21.09 (10.01)	46	23.12 (11.00)	41	22.02 (10.95)	51	22.61 (10.19)	51
BSI*		-		0.65 (.52)	-		-		-		-		-	
	ART	1.29 (0.62)	68										0.93 (0.69)	53
	CFC	1.23 (0.63)	54										0.98 (0.59)	50
	WLC	1.16 (0.68)	54										1.04 (0.64)	52
ERSQ		-		0.41 (.66)										
	ART	1.72 (0.66)	66		1.85 (0.71)	66	2.07 (0.77)	62	2.19 (0.72)	58	2.18 (0.65)	52	2.23 (0.71)	53
	CFC	1.73 (0.63)	51		1.73 (0.63)	57	1.85 (0.70)	57	2.09 (0.68)	46	1.91 (0.77)	43	2.05 (0.81)	50
	WLC	1.63 (0.62)	52		1.69 (0.66)	56	1.74 (0.58)	45	1.77 (0.71)	41	1.71 (0.66)	53	1.74 (0.64)	52
ERSQ-OB		-		0.25 (.98)	-		-		-		-		-	
	ART	2.09 (0.72)	48										2.61 (0.69)	39
	CFC	2.06 (0.62)	47										2.30 (0.67)	31
	WLC	2.09 (0.71)	37										2.03 (0.67)	35

ART = Affect Regulation Training; CFC = Common Factor Control Condition; WLC = Waitlist Control Condition

BSI* = BSI without depression scale.

**Table 3:** Simple slopes, cross-level interaction effects and pairwise comparisons between groups for depression, comorbidity, and emotion regulation total scores.

Outcome	Effect/Groups	Estimate	SE	df	95% CI	t / χ^2^
HRSD/BDI-II						
	Time: ART	-0.78	0.10	188.83	-0.97 –-0.58	
	Time: CFC	-0.52	0.10	191.20	-0.73 –-0.32	
	Time: WLC	-0.22	0.11	190.85	-0.43 –-0.01	
	Time x group (χ^2^)			2		14.90***
	ART vs. WLC (t)	-0.56	0.14	188.19	-0.84 –-0.27	-3.86***
	ART vs. CFC (t)	-0.25	0.14	188.25	-0.53–0.03	-1.76
	CFC vs. WLC (t)	-0.30	0.15	189.70	-0.59 –-0.01	-2.05^a^
ERSQ						
	Time: ART	0.78	0.14	165.04	0.51–1.05	
	Time: CFC	0.51	0.14	167.30	0.22–0.79	
	Time: WLC	0.05	0.14	163.44	-0.23–0.33	
	Time x group (χ^2^)			2		14.27***
	ART vs. WLC (t)	0.73	0.20	164.21	0.35–1.12	3.75***
	ART vs. CFC (t)	0.27	0.20	166.23	-0.11–0.66	1.39
	CFC vs. WLC (t)	0.46	0.20	165.38	0.07–0.85	2.29^a^
ERSQ-OB						
	Time: ART	0.60	0.14	97.37	0.32–0.88	
	Time: CFC	0.33	0.16	103.38	0.02–0.65	
	Time: WLC	-0.09	0.16	95.44	-0.40–0.22	
	Time x group (χ^2^)			2		10.83**
	ART vs. WLC (t)	0.69	0.21	90.69	0.28–1.11	3.28**
	ART vs. CFC (t)	0.27	0.21	94.92	-0.14–0.69	1.26
	CFC vs. WLC (t)	0.42	0.22	93.71	-0.01–0.85	1.91^a^
BSI*						
	Time: ART	-0.50	0.10	143.48	-0.69 –-0.30	
	Time: CFC	-0.33	0.11	148.65	-0.55 –-0.12	
	Time: WLC	-0.17	0.11	144.72	-0.37–0.04	
	Time x group (χ^2^)			2		5.38
	ART vs. WLC (t)	-0.33	0.14	144.94	-0.62 –-0.05	-2.32*
	ART vs. CFC (t)	-0.17	0.15	147.10	-0.45–0.12	-1.15
	CFC vs. WLC (t)	-0.17	0.15	147.53	-0.46–0.13	-1.10

ART = Affect Regulation Training; CFC = Common Factor Control Condition; WLC = Waitlist Control Condition. OB = Observer-based.

* p < .05

** p < .01

*** p < .001

^a^ p < .10

BSI* = BSI without depression scale.

### Differences in change of depressive symptoms

Consistent with our primary hypothesis, HLM-based analyses show that depressive symptom reduction was significantly stronger in the ART than in the WLC condition (p < .001; see Table 3). A controlled effect size of d = 0.56 indicated moderate treatment effects. These effects were not moderated by comorbidities (exclusively meeting criteria for MDD vs. meeting criteria for at least one other mental disorder), χ^2^ (2) = 0.75, p = .69; nor were they moderated by missingness (completed all assessments vs. missed at least one assessment), χ^2^ (2) = 0.47, p = .63. Further analyses indicated that in the ART group, 27.4% / 29.6% of participants met the criteria for remission according to HRSD / BDI-II, respectively, whereas in the WLC group, only 16.7% (HRSD) and 17.6% (BDI-II) met the criteria for remission. There were no significant differences between ART and CFC (d = 0.25 in favor of ART, p = .19), nor between CFC and WLC (d = 0.3 in favor of CFC, p = .10) with regard to the reduction of DSS. The rate of remission in CFC was 24.6% / 24% according the HRSD / BDI-II criteria, respectively.

### Differences in secondary outcomes

The findings shown in Table 3 indicate that there was a significantly greater increase in self-reports on adaptive responses to undesired feelings in ART than in WLC, ERSQ_total score_: d = 0.73, p < .001. Observer-based ratings of emotion regulation consistently indicate that ART is associated with a greater increase of emotion regulation skills than WLC is, ERSQ-OB_total score_: d = 0.69, p < .01. With regard to the subscales of the ERSQ (see Table 4), the greatest differences were found for self-support (d = 0.85), modification (d = 0.78), *acceptance* (d = 0.58), and *tolerance* (d = 0.53). For the ERSQ-OB, the greatest between-group differences of change resulted in *understanding* (d = 0.80), *acceptance* (d *=* 0.71), *self-support* (d = 0.64), and *modification* (d *=* 0.62). Participants in ART also reported a significantly greater reduction of psychopathological symptoms other than depression as assessed with the BSI*, d = 0.33, p < .02, than did participants in WLC.

**Table 4:** Cross-level interaction effects and pairwise comparisons between groups for emotion regulation subscales measures.

Outcome	*B*		SE		df		95% CI		t / χ^2^	
ERSQ–ERSQ-OB	self	other	self	other	self	other	self	other	self	other
Awareness										
Time x group (χ^2^)					2	2			6.26*	7.96*
ART vs. WLC (t)	0.45	0.56	0.18	0.20	157.57	93.38	0.10–0.81	0.17–0.96	2.49*	2.77**
ART vs. CFC (t)	0.17	0.35	0.18	0.20	159.45	98.45	-0.18–0.53	-0.04–0.76	0.95	1.74^a^
CFC vs. WLC (t)	0.28	0.21	0.19	0.21	158.72	96.62	0.09–0.64	-0.21–0.62	1.49	0.97
Sensations to identify emotions										
Time x group (χ^2^)					2	2			5.65^a^	4.17
ART vs. WLC (t)	0.41	0.43	0.18	0.26	168.09	104.30	0.06–0.77	-0.09–0.95	2.28^a^	1.61
ART vs. CFC (t)	0.09	-0.10	0.18	0.27	170.00	111.38	-0.27–0.45	-0.62–0.42	0.48	-0.39
CFC vs. WLC (t)	0.33	0.53	0.19	0.28	169.20	110.56	-0.03–0.70	-0.01–1.07	1.75^a^	1.91^a^
Clarity										
Time x group (χ^2^)					2	2			10.80**	5.54^a^
ART vs. WLC (t)	0.33	0.51	0.18	0.23	168.94	88.13	-0.03–0.68	0.07–0.97	1.81	2.23*
ART vs. CFC (t)	-0.28	0.08	0.18	0.23	170.95	94.78	-0.64–0.08	-0.37–0.53	-1.54	0.33
CFC vs. WLC (t)	0.61	0.44	0.19	0.24	169.89	93.45	0.25–0.97	-0.03–0.90	3.28**	1.80^a^
Understanding										
Time x group (χ^2^)					2	2			6.73*	10.79**
ART vs. WLC (t)	0.47	0.80	0.21	0.24	173.83	95.86	0.06–0.89	0.32–1.28	2.25^a^	3.27**
ART vs. CFC (t)	-0.02	0.30	0.21	0.24	175.79	103.09	-0.43–0.40	-0.18–0.77	-0.07	1.21
CFC vs. WLC (t)	0.49	0.50	0.22	0.26	175.01	101.63	0.07–0.91	0.00–1.00	2.26^a^	1.96*
Acceptance										
Time x group (χ^2^)					2	2			7.00*	8.95**
ART vs. WLC (t)	0.58	0.71	0.22	0.24	166.29	90.56	0.14–1.01	0.25–1.18	2.61*	2.98**
ART vs. CFC (t)	0.18	0.28	0.22	0.24	168.30	97.10	-0.25–0.62	-0.18–0.74	0.82	1.56
CFC vs. WLC (t)	0.39	0.43	0.23	0.25	167.65	96.40	-0.05–0.84	-0.05–0.92	1.73	1.74a
Tolerance										
Time x group (χ^2^)					2	2			6.80*	3.76
ART vs. WLC (t)	0.53	0.47	0.21	0.24	159.02	101.76	0.13–0.94	-0.01–0.95	2.59*	1.91
ART vs. CFC (t)	0.32	0.29	0.21	0.25	161.07	109.44	-0.09–0.72	-0.19–0.77	1.53	1.18
CFC vs. WLC (t)	0.22	0.17	0.21	0.26	160.30	107.59	-0.20–0.63	-0.33–0.67	1.02	0.67
Readiness to confront										
Time x group (χ^2^)					2	2			5.41^a^	6.97*
ART vs. WLC (t)	0.33	0.47	0.16	0.25	770.39	97.43	0.01–0.64	-0.01–0.96	2.01*	1.89^a^
ART vs. CFC (t)	0.32	-0.19	0.16	0.25	769.13	104.94	0.00–0.64	-0.68–0.29	1.98*	-0.77
CFC vs. WLC (t)	0.00	0.67	0.17	0.26	772.86	102.64	-0.32–0.33	0.16–1.17	0.01	2.56**
Self-support										
Time x group (χ^2^)					2	2			19.84***	7.01*
ART vs. WLC (t)	0.85	0.64	0.19	0.25	158.42	100.34	0.47–1.23	0.15–1.13	4.36***	2.56**
ART vs. CFC (t)	0.56	0.44	0.19	0.25	160.43	107.18	0.18–0.94	-0.05–0.92	2.86**	1.74^a^
CFC vs. WLC (t)	0.29	0.20	0.19	0.26	159.92	106.16	-0.10–0.68	-0.31–0.71	1.45	0.77
Modification										
Time x group (χ^2^)					2	2			16.51***	7.68*
ART vs. WLC (t)	0.78	0.62	0.19	0.25	538.10	95.35	0.40–1.15	0.14–1.11	4.06***	2.52**
ART vs. CFC (t)	0.36	0.54	0.19	0.25	541.58	101.91	-0.02–0.74	0.05–1.02	1.87	2.16*
CFC vs. WLC (t)	0.42	0.09	0.20	0.26	548.55	101.17	0.03–0.80	-0.42–0.59	2.13^a^	0.33

ART = Affect Regulation Training; CFC = Common Factor Control Condition; WLC = Waitlist Control Condition.

* p < .05

** p < .01

*** p < .001

^a^ p < .10.

Compared with the effects of CFC, the effects of ART regarding the increase in adaptive responses toward undesired affective states did not reach critical alpha (ERSQ_total score_: p = .15; ERSQ-OB_total score_: p = .21, with effect sizes (ERSQ_total score_: d = 0.27, ERSQ-OB_total score_: d = 0.27), corresponding to small effects according to Cohen (1988). Similarly, no significant differences between ART and CFC were found with regard to the reduction of psychopathological symptoms other than depression (BSI*: d = 0.17, p = .25). When comparing CFC with WLC, the results indicated that adaptive responses toward undesired feelings assessed by self-report increased to a greater extent in CFC than in WLC (ERSQ: d = 0.46, p < .10). For psychopathological symptoms other than depression, there were no significant differences between CFC and WLC (d = 0.17, p = .27).

### Mediation analysis

**Fig 2:**
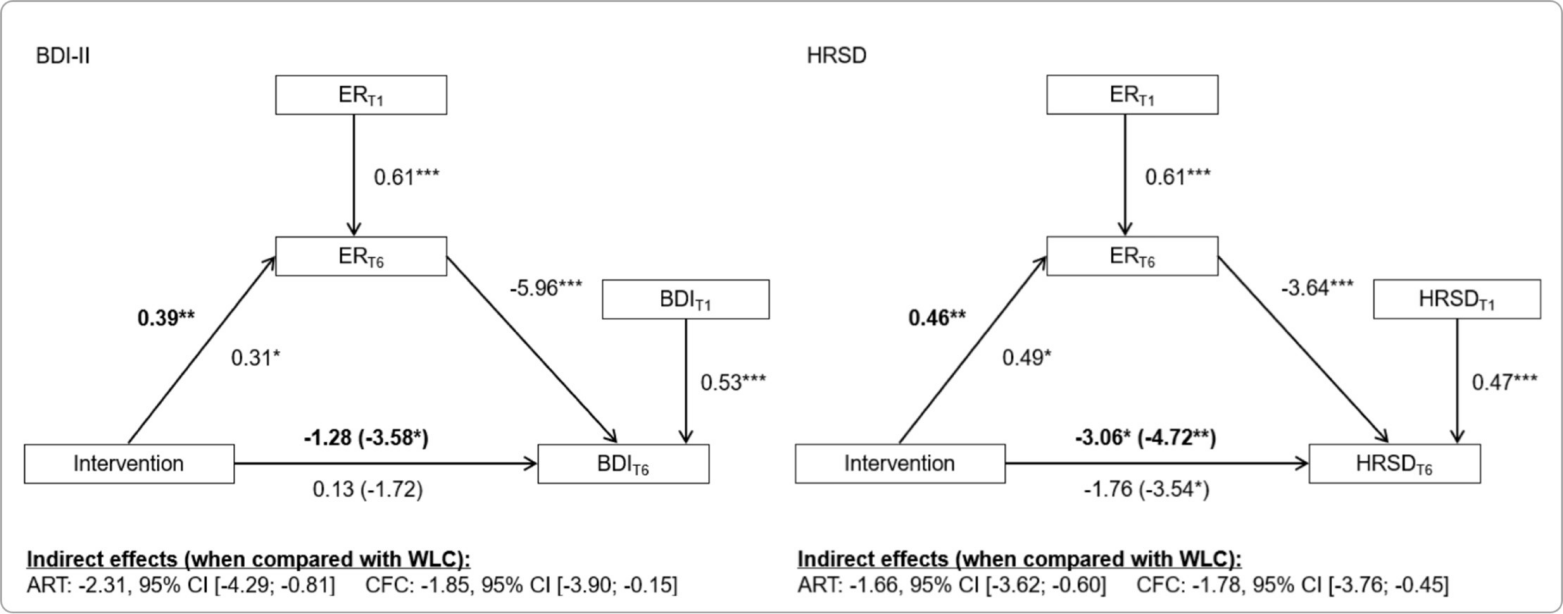
Affect regulation mediates the effects of both active treatments on depression. ER = emotion regulation; BDI-II = Beck Depression Inventory II; HRSD = Hamilton Rating Scale for Depression; *p < .05, **p < .01, ***p < .001.

The results of the mediation analyses are illustrated in Fig 2. Consistent across both measures of depression, participants in the ART and CFC groups had significantly higher post-treatment levels of emotion regulation skills than did participants in the WLC group, controlling for pre-treatment levels of emotion regulation. Higher post-treatment levels of these skills were negatively associated with lower post-treatment levels of depressive symptoms, controlling for pre-treatment levels of depressive symptoms. For both outcomes, the indirect effect was significant in both the ART group and the CFC group. These findings indicate that both ART and CFC are effective in reducing depressive symptoms by enhancing participants’ emotion regulation skills.

## Discussion

The primary aim of the present study was to evaluate the efficacy of a training of general emotion regulation skills with regard to reducing depressive symptoms in individuals meeting criteria for MDD. To achieve this aim, we conducted a randomized controlled trial with N = 218 clinically depressed participants, comparing the rates of symptom reduction among participants assigned to the group-based Affect Regulation Training (ART), a waitlist control condition (WLC), or a group-based treatment primarily developed to control for common factors of psychological interventions (CFC). The main findings of the study indicate that ART is superior to WLC with regard to reducing DSS, enhancing emotion regulation skills, and reducing psychopathological symptoms other than depression.

Thus, with regard to the goal of enhancing our understanding of the role of emotion regulation in psychopathology, this study contributes to the literature by showing that experimentally manipulating emotion regulation skills in the field (with the help of an intervention that focuses exclusively on strengthening adaptive responses toward undesired feelings) was significantly associated with changes in DSS in clinically depressed patients. As such, the findings go beyond previous findings from observational studies (e.g., [64]), laboratory-based findings (e.g., [63]), outcome studies evaluating treatments that focus on emotion regulation as well as other skill domains (e.g., [26]), and outcome studies in which a transdiagnostic emotion regulation training was used as an adjunctive intervention [2, 66].

Another interesting finding from the present study is that CFC also led to significant improvement of emotion regulation skills (although more sophisticated ways of utilizing common factors can be imagined (e.g. [113])). This finding indicates that such abilities can be fostered not only by systematically practicing skills that directly focus on responses toward undesired emotions (as in ART) but also by focusing on the misfit between motivation and perception as a significant antecedent of affective responses. Consistent with this hypothesis, CFC also led to a significant reduction of depressive symptoms. Moreover, the results from the mediation analyses indicate that the increase of emotion regulation skills is an important mechanism of change in both ART and CFC. These findings are in line with research indicating that various evidence-based treatments for mental disorders reduce maladaptive emotion regulation [5]. The findings significantly add to the literature because of the (regrettably rare) use of mediation analyses in randomized clinical trials and because of the use of identical measures of emotion regulation in all conditions (currently, findings on emotion regulation and psychopathology are often difficult to compare because they use different definitions or measures of emotion regulation [5]). Thus, the present study provides evidence for the assumption raised by some authors (e.g., [114]) that enhancing emotion regulation skills might be not only a promising target in the treatment of various disorders but also a common factor promoting change in various evidence-based treatments. If future studies provide further evidence in support of this hypothesis, research should attempt to clarify whether including the enhancement of emotion regulation into common factor approaches to psychotherapy (e.g., [115]) may help to increase the heuristic/therapeutic value of these approaches. For example, training therapists to recognize moments in treatment in which fostering adaptive emotion regulation is most helpful and to apply a broad range of interventions shown to foster emotion regulation may facilitate the proper balance of the use of standardized interventions and the flexible adaptation of the treatment to the specific needs, strengths and motivation of the individual patient.

To determine the implications of our findings for clinical practice, it is necessary to clarify how the effects of ART on depression compare to those of disorder-specific treatments for depression. In a recent meta-analysis primarily including studies on individual therapy for depression, Mohr and colleagues [116] reported an average effect size of *g* = 0.95 for comparisons with waiting control conditions. Another meta-analysis found that individual disorder-specific therapies for depression are significantly more effective than group-based treatments (with d = 0.20 between these two settings [117]). When using these results as benchmarks, ART appears less effective than individual disorder-specific treatment and less effective than group-based disorder-specific treatments. However, this conclusion is preliminary because (i) the treatment periods in these studies were notably longer than those investigated in the present study, (ii) there is evidence from meta-analytical findings that aggregated effect sizes are distorted by publication biases [116, 118], (iii) the effect sizes reported in a meta-analysis of the efficacy of disorder-specific treatments for depression found an aggregated effect size of d = 0.53 when outliers were excluded from the analyses [119], (iv) effect sizes reported for high-quality studies (defined by features they shared to a significant extent with the present study) have been reported as low as d = 0.24 for individual and d = 0.20 for group-based treatments for depression [118], and (v) there is evidence that the effect sizes of studies comparing bona fide disorder-specific treatments for depression with waitlist control conditions can also be lower than those found in the present study [120]. Nevertheless, the present study provides significant proof of the principle that by systematically enhancing emotion regulation skills, depressive symptom severity can be reduced in individuals with MDD. The present study provides no evidence that a comparatively brief, group-based, transdiagnostic emotion regulation skills training would be superior to current disorder-specific treatments. The comparatively small percentage of patients remitted after the six plus one group sessions provided in ART indicates that a large number of patients require further treatment. Future research should clarify to what extent the remission rate can be further enhanced by extending and intensifying transdiagnostic emotion regulation trainings.

Another question relevant to clinical practice is how ART relates to other transdiagnostic approaches targeting depression (and other disorders). The most important distinction is that ART focuses exclusively on how to respond to undesired emotion without providing any information or training on how to cope with specific mental disorders. This feature distinguishes ART from treatments such as the UP, Cognitive Behavioral Therapy for Eating Disorders [32], Transdiagnostic Behavior Therapy for Veterans with Affective Disorders [121–123], Transdiagnostic Cognitive Behavioral Group Therapy [124], or Emotion Regulation Therapy [36, 37], all of which focus on particular subgroups of (commonly co-occurring) disorders. Because of its entirely transdiagnostic format, ART has been proposed as a (stand-alone) intervention that can be used when enhancing emotion regulation skills alone is expected to suffice for preventing or coping with a mental disorder or (used as an adjunctive intervention) when strengthening the focus on emotion regulation skills appears promising to overcome a mental disorder. This proposal is based on the following assumptions: (i) any kind of cognitive or behavioral activity that plays a role in maintaining a mental disorder may initially *reduce* negative effects (to some extent); (ii) individuals experiencing negative emotions are tempted to engage in pathogenic cognitive or behavioral processes when they encounter negative affective states; (iii) engaging in pathogenic processes is reinforced by the subsequent (short-term) reduction of negative mood; and (iv) the likelihood of engaging in these pathogenic processes is reduced by providing adaptive ways of responding to undesired affective states (which ideally reduce negative affect in both a short- and long-term perspective) [22]. Empirical support for this line of argument comes from the rapidly growing number of studies associating various forms of mental disorders with deficits in emotion regulation (e.g., [1, 5, 20]). Given preliminary evidence that emotion regulation skills can be enhanced with transdiagnostic trainings [2, 66, 82, 125], there is significant support for the hypothesis that strengthening emotion regulation skills might be a promising approach to treating mental disorders *in general*.

Major limitations of the present study include the exclusive focus on individuals meeting criteria for MDD. The potential contribution of ART to clinical practice is based on the fact that ART was developed to strengthen adaptive responses to any kind of negative affect without addressing the characteristics of specific mental disorders. From this, it can be concluded that ART can be *applied* to any kind of mental disorder. However, this does not mean that ART is *effective* in treating any kind of mental disorder. Thus, ideally, the present study should have included enough patients for each disorder (or at least for the most common/important disorders) listed in the DSM-5 to provide sufficient statistical power to evaluate the efficacy of ART both as a stand-alone and as an add-on intervention in comparison to a waitlist and an active control condition for each of these disorders.

Another major limitation of the study is the lack of a comparison between ART and the gold standard treatment of depression. Given the abundant evidence for the importance of disorder-specific maintenance factors (e.g., [126]), an exclusive focus on emotion regulation is unlikely to have effects that are comparable with those attained by gold standard treatments. However, because of the potential advantage regarding cost-efficacy or additional effects on multiple/comorbid disorders, future studies should attempt to quantify the difference between transdiagnostic emotion regulation trainings and gold standard treatments with respect to cost-efficacy and comorbid symptom reduction. Ideally, such studies should compare both individual- and group-based formats of transdiagnostic and disorder-specific treatments in patients who differ with regard to the number of disorders they meet criteria for. Moreover, although interventions such as ART may not be the most effective stand-alone intervention for specific disorders, (some) first-line treatments might benefit from a stronger focus on emotion regulation [66]. Thus, future studies should also work to identify populations/disorders for which the integration of interventions such as ART may help to further improve efficacy (e.g., [127]).

Further limitation of the present study include the lack of a long-term follow-up (because all participants received individual CBT for depression to evaluate the potential augmenting effects of ART on subsequent individual therapy), the lack of data on adherence in a way that would allow for statistically relating adherence to outcome, and the lack of explicit assessment of adverse effects. Thus, it is presently unclear whether the differences found across groups would be stable of longer periods of time, whether the outcome is related to adherence, and to what extent adverse effects occurred in the treatment conditions.

With regard to the latter, it is of note that we systematically looked for adverse effects when supervising therapists in the active conditions and found none. Additionally, when we asked participants allocated to one of the active conditions who dropped out of the study because of the reported inability to cope with the additional strain (n = 6; see Fig 1) associated with the study to specify the nature of this strain, we unanimously received the answer that the strain was caused by the extensive assessment battery and not by the treatment. Finally, it is of note that there was an above-average percentage of strain related drop-out in the waitlist control condition. Therefore, we presently assume that there are no specific adverse effects associated with the two active treatments.

Nevertheless, future studies should assess and evaluate adverse effects with standardized instruments. In addition to including follow-up assessments and adherence ratings for all treatments, these studies should also evaluate to what extent emotion regulation trainings can be used in later stages of treatment or as aftercare interventions to prevent patients from relapsing when confronted with undesired affective states [57].

## Supporting information

S1 File CONSORT checklist. (DOC)
